# Further Insights into the Ciliary Gene and Protein KIZ and Its Murine Ortholog PLK1S1 Mutated in Rod-Cone Dystrophy

**DOI:** 10.3390/genes8100277

**Published:** 2017-10-18

**Authors:** Said El Shamieh, Cécile Méjécase, Matteo Bertelli, Angélique Terray, Christelle Michiels, Christel Condroyer, Stéphane Fouquet, Maxime Sadoun, Emmanuelle Clérin, Binqian Liu, Thierry Léveillard, Olivier Goureau, José-Alain Sahel, Isabelle Audo, Christina Zeitz

**Affiliations:** 1 Sorbonne Universités, UPMC University Paris 06, INSERM U968, CNRS UMR 7210, Institut de la Vision, 75012 Paris, France; said.shamieh@gmail.com (S.E.S.); cecile.mejecase@inserm.fr (C.M.); angelique.terray@inserm.fr (A.T.); christelle.michiels@inserm.fr (C.M.); christel.condroyer@inserm.fr (C.C.); stephane.fouquet@inserm.fr (S.F.); m.sadoun@hotmail.fr (M.S.); emmanuelle.clerin@inserm.fr (E.C.); emilie.cy.liu@gmail.com (B.L.); thierry.leveillard@inserm.fr (T.L.); olivier.goureau@inserm.fr (O.G.); j.sahel@gmail.com (J.-A.S.); 2 Department of Medical Laboratory Technology, Faculty of Health Sciences, Beirut Arab University, 115020 Beirut, Lebanon; 3 Magi Euregio, 39100 Bolzano (BZ), Italy; bertellimatteo@hotmail.com; 4 Centre Hospitalier National d’Ophtalmologie des Quinze-Vingts, DHU Sight Restore, INSERM-DHOS CIC 1423, 75012 Paris, France; 5 Fondation Ophtalmologique Adolphe de Rothschild, 75019 Paris, France; 6 Académie des Sciences-Institut de France, 75006 Paris, France; 7 Department of Ophthalmology, The University of Pittsburgh School of Medicine, Pittsburg, PA 15213, USA; 8 Institute of Ophthalmology, University College of London, London EC1V 9EL, UK

**Keywords:** rod-cone dystrophy, mouse retina, KIZ, PLK1S1, functional characterization

## Abstract

We identified herein additional patients with rod-cone dystrophy (RCD) displaying mutations in *KIZ*, encoding the ciliary centrosomal protein kizuna and performed functional characterization of the respective protein in human fibroblasts and of its mouse ortholog PLK1S1 in the retina. Mutation screening was done by targeted next generation sequencing and subsequent Sanger sequencing validation. *KIZ* mRNA levels were assessed on blood and serum-deprived human fibroblasts from a control individual and a patient, compound heterozygous for the c.52G>T (p.Glu18*) and c.119_122del (p.Lys40Ilefs*14) mutations in *KIZ*. KIZ localization, documentation of cilium length and immunoblotting were performed in these two fibroblast cell lines. In addition, PLK1S1 immunolocalization was conducted in mouse retinal cryosections and isolated rod photoreceptors. Analyses of additional RCD patients enabled the identification of two homozygous mutations in *KIZ*, the known c.226C>T (p.Arg76*) mutation and a novel variant, the c.3G>A (p.Met1?) mutation. Albeit the expression levels of *KIZ* were three-times lower in the patient than controls in whole blood cells, further analyses in control- and mutant *KIZ* patient-derived fibroblasts unexpectedly revealed no significant difference between the two genotypes. Furthermore, the averaged monocilia length in the two fibroblast cell lines was similar, consistent with the preserved immunolocalization of KIZ at the basal body of the primary cilia. Analyses in mouse retina and isolated rod photoreceptors showed PLK1S1 localization at the base of the photoreceptor connecting cilium. In conclusion, two additional patients with mutations in *KIZ* were identified, further supporting that defects in KIZ/PLK1S1, detected at the basal body of the primary cilia in fibroblasts, and the photoreceptor connecting cilium in mouse, respectively, are involved in RCD. However, albeit the mutations were predicted to lead to nonsense mediated mRNA decay, we could not detect changes upon expression levels, protein localization or cilia length in *KIZ*-mutated fibroblast cells. Together, our findings unveil the limitations of fibroblasts as a cellular model for RCD and call for other models such as induced pluripotent stem cells to shed light on retinal pathogenic mechanisms of *KIZ* mutations.

## 1. Introduction

Rod-cone dystrophy (RCD), also known as retinitis pigmentosa, is a heterogeneous group of inherited retinal disorders affecting rod photoreceptors in the majority of cases with secondary cone degeneration [1]. Population-based studies showed that RCD affects one in 4000 individuals around the world [1]. Subjects diagnosed with RCD initially complain of night blindness followed by progressive visual field constriction, abnormal color vision and eventually loss of central vision [1]. These visual symptoms indicate the gradual loss of the two photoreceptor types: rods, which mediate achromatic vision in dim-lit environments and cones, which are important for daylight color vision and fine acuity [1]. Photoreceptors are post-mitotic sensory neurons that display a polarized structure including a biosynthetically-active inner segment (IS) and a photosensitive outer segment (OS) [2]. The two segments are connected via a specialized bridge-like structure, the connecting cilium (CC), which represents the transitional zone of a prototypical primary cilium [2]. The CC is a region with increasing relevance for ciliary homeostasis due to its implication in ciliogenesis [3] and protein trafficking [4].

RCD is inherited as a Mendelian trait in most cases [1]. On the basis of its mode of inheritance and prevalence, RCD can be divided into three groups: autosomal dominant (ad) (15–25%), autosomal recessive (ar) (35–50%) and X-linked (xl) (7–15%) [5]. To date, mutations in more than 83 genes are implicated in non-syndromic RCD [6,7] Of those, at least 15 encode proteins that localize to the CC and/or basal body (BB) of the cell [8]. Most of the RCD mutant genes account for only a small proportion of cases, with the exception of rhodopsin (*RHO*, Mendelian Iheritance in Man (MIM): 180380), usherin (*USH2A*, MIM: 608400) and retinitis pigmentosa GTPase regulator (*RPGR*, MIM: 312610) in which mutations together account for ~30% of all RCD cases [1,6].

Our group has previously identified mutations in a novel gene *KIZ* (MIM: 615757), encoding a ciliary centrosomal protein, kizuna. The precise function of this protein, whose defect accounts for 1% of arRCD in the European population [9], is still unknown. The purpose of the current study was to report additional mutations in *KIZ* and further characterize its protein localization.

## 2. Materials and Methods

### 2.1. Ethics Statement

The study protocol was conducted in accordance with the declaration of Helsinki, the national guidelines and the regional ethics committee. Informed written consent was obtained from every participant. Human DNA collection for genetic research was project No. EUDRACT 2006-A00347-44, n°06693 and was approved by CPP Ile de France V (regional ethics committee) on the 24th of October 2006. Human skin biopsies to derive cells for research was project n°10820 and was approved by the CPP Ile de France V on the 8th of November 2010 and expanded on the 2nd of October 2012.

All animal procedures were performed according to the Association for Research in Vision and Ophthalmology (ARVO) Statement for the Use of Animals in Ophthalmic and Visual Research and were approved by the French Minister of Agriculture (authorization A-75-1863 delivered on 9 November 2011). All efforts were made to minimize suffering.

### 2.2. Targeted Next-Generation Sequencing and Validation

The next generation sequencing (NGS) panel was selected from the SureSelect Human All Exon Kits Version 4 (Agilent, Massy, France) and covers 198 IRD genes in total. The eArray web-based probe design tool that was used for this purpose can be found at https://earray.chem.agilent.com/earray. All probes were designed and synthesized by Agilent Technologies (Santa Clara, CA, USA). Sequence capture, enrichment and elution were performed according to Agilent’s instructions. Identified variants in *KIZ* (NM_018474.4) were validated by direct Sanger sequencing, and co-segregation analyses was performed in the DNA of available family members as previously described [9]. Similarly, the presence of the two mutations c.52G>T, p.Glu18* and c.119_122del, p.Lys40Ilefs*14 in the fibroblast cell line of patient CIC01225 was confirmed by direct Sanger sequencing using genomic primers and conditions as previously described [9].

### 2.3. Pathogenic Predictions

To assess the pathogenicity of the novel *KIZ* variant, we studied amino acid conservation across 30 species in the UCSC Genome Browser (University of California, Santa Cruz, CA, USA), for which a coding sequence was present [10]. Pathogenicity was also evaluated on the basis of bio-informatic predictions as Polyphen (polymorphism phenotyping) [11] and SIFT (sorting intolerant from tolerant) [12]. In addition, we determined the exact minor allele frequency of the herein identified variants in *KIZ* with the Genome Aggregation Database (gnomAD) [13] or a software (Alamut v2.7.1, Interactive Biosoftware, Rouen, France).

### 2.4. RNA Extraction and Real Time PCR Analysis

In total, we investigated four whole blood samples, among which three were controls and one corresponding to the affected individual: CIC01225 (compound heterozygous for the c.52G>T (p.Glu18*) and c.119_122del (p.Lys40Ilefs*14) mutations in *KIZ*). Two samples of human fibroblasts (unaffected vs. CIC01225) were also included. Total RNA was isolated from whole blood cells and fibroblasts of CIC01225, Family C (737), using a kit (RNeasy Mini Kit, Qiagen, Hilden, Germany), and 500 ng were used to synthesize cDNA with a reverse transcriptase (SuperScript^®^II, Invitrogen, Carlsbad, Czech Republic), according to the manufacturer’s protocol. Quantitative real-time polymerase chain reaction (PCR) was performed using a PCR system (Applied Biosystems 7500 Real-Time, Life technologies) with a kit (Power SYBR Green kit, Applied Biosystems, Life technologies) for *KIZ* and 18S transcripts. Specific primers were designed in exons 6 and 7 of *KIZ* (6F: 5′-CAGAACCACAGCCAAATCCA-3′; and 7R: 5′-TCTGAGTCTGGTGTTTGTCC-3′) spanning intron 6. All experiments were carried out in triplicates in a total reaction volume of 25 µL containing 2.5 ng of cDNA. *KIZ* mRNA levels were normalized to 18S rRNA. To test for the expression of the different exons of *KIZ* in blood, fibroblasts and retina, PCR-experiments on cDNA were performed with primers located in exons 1–4 (1F: 5′-GTCTCCTTCGGCAACCCC-3′; and 4R: 5′-TGTCTTCATCTGTCAGTTCCTC -3′), in exons 4–6 (4F: (5′-GAGGAACTGACAGATGAAGAC-3′; and 6R: 5′-CCTGGATTTGGCTGTGGTTC-3′) and in exons 6–13 (6F: (5′-CAGAACCACAGCCAAATCCA-3′; and 13R: 5′-AGTTACTGTCATCAGACTCATCC-3′). All experiments were carried out in a total reaction volume of 12.5 µL using 3 µL of 1:10 diluted cDNA. The specificity of all PCR products was first verified by electrophoresis on 2% agarose gel and subsequently by Sanger sequencing.

### 2.5. Statistical Analysis

Statistical analyses were conducted using SPSS software Version 20 (SPSS, Inc., Chicago, IL, USA). Since normalized *KIZ* expression and cilium length data are arbitrarily distributed, we used a Mann–Whitney *U* test, a non-parametric test that compares the medians. This is the reason behind representing the data in a box plot format where the median (bar), the maximum and minimum values and the quartiles are shown. To investigate whether the compound mutation in *KIZ* decreased the percentage of ciliated cells, we performed a chi-square test using two categorical variables: ciliated cell (yes and no) vs. individual (control and CIC01225) in ~60 fibroblast cells equally divided as control and affected. The threshold for statistical significance was set at a *p*-value of <0.05.

### 2.6. Antibodies and Microscopy

Anti-acetylated α-tubulin mouse monoclonal antibody (T7451), anti-γ-tubulin mouse monoclonal antibody (T6557) and anti-c-myc mouse monoclonal antibody (11667149001) were purchased from companies (Sigma-Aldrich, St. Quentin Fallavier, France for the first two; Roche, Basel, Switzerland, for the last). Mouse monoclonal anti-rhodopsin antibody (MAB5316) and Lectin PNA conjugate 594 (L-32460) were purchased from two other companies (Millipore, Guyancourt, France and Invitrogen, respectively). Secondary donkey antibodies anti-rabbit and anti-mouse were conjugated with Alexa Fluor 488 (711-545-152, Jackson Immuno Research Laboratories, Baltimore, MD, USA) and Cy3 (715-165-150, Jackson Immuno Research) or with Alexa Fluor 647 (715-605-150, Jackson Immuno Research) fluorophores, respectively. The 40,6-diamidino-2-phenylindole (DAPI) was purchased from a company (Euromedex, Souffelweyersheim, France). In addition, anti-mouse and anti-rabbit horse radish peroxidase (HRP)-antibodies (715-035-150 and 711-545-152, respectively) (Jackson Immuno Research Baltimore, MD) were used for Western blot analysis. Confocal image stacks were performed on an Olympus FV1200 laser-scanning confocal microscope (Olympus, Rungis, France). DAPI counterstaining, AlexaFluor-594 and AlexaFluor-488 immunolabeling were excited by using 405- and 559-nm laser diodes lines and 488–515-nm argon ion laser lines, respectively. The objectives used was an Olympus 40x NA1.30-WD 0.20 or PLAPON 60x SC NA 1.40 WD 0.12. Control of the microscope and image acquisition was conducted by using Olympus Fluoview software Version 4.2. Image acquisition was conducted at a resolution of 1024 × 1024 pixels, with a scan rate of 8–10  μs·pixel^−1^ and with or without zoom. Images were acquired sequentially, line by line, in order to reduce excitation and emission crosstalk, and step size was defined according to the Nyquist–Shannon sampling theorem. Exposure settings that minimized oversaturated pixels in the final images were used. Twelve-bit images were processed with ImageJ or FIJI (National Institutes of Health, Bethesda, MD, USA) and finally converted in 24-bit RGB color mode. Figures were then assembled by using the Adobe Photoshop CC and Adobe Illustrator CC softwares (Adobe Systems, Mountain View, CA, USA).

### 2.7. Antibody Tests

The human KIZ and its mouse ortholog PLK1S1 are 66% identical. Therefore, we used the following species-specific antibodies. The anti-PLK1S1 antibody, which was a gift of Blanche Capel (Duke University Medical Center, Durham, NC, USA) [14], is a rabbit polyclonal antibody produced against the 3’ end of mouse PLK1S1. The ability of this antibody to detect PLK1S1 was demonstrated in the work of Tang et al. [14]. At that time, murine *Plk1s1* was called *Gm114*. Using the Western blot experiment on adult wild-type mouse testis lysates, anti-PLK1S1 recognized a 76-kDa protein, the predicted size of the full-length protein [14], which was absent in homozygous mutants. In addition, the GM114 protein was also not detected by immunocytochemistry in the mutant adult testis, while it shows a nice immunolocalization in wild-type mice [14]. Therefore, we considered this antibody to be able to detect PLK1S1.

The anti-KIZ antibody is a commercially available rabbit polyclonal antibody (SAB2700541, Sigma-Aldrich) produced with a recombinant human *KIZ* fragment corresponding to the region between amino acids 187 and 438 of the human protein. To check if it is indeed able to detect the human protein, we overexpressed a c-myc tagged *KIZ* construct in COS-1 cells. In detail: a pBudCE4.1 plasmid (GeneCust, Dubelange, Luxembourg) containing coding exons of the human *KIZ* sequence (reference NM_018474.4) tagged with the c-myc epitope was synthesized. To test if the anti-KIZ antibody is indeed able to detect the respective protein by immunolocalization studies, the following procedures were performed:

In 24-well plates, 175,000 COS-1 cells per well were seeded on coated coverslips and transfected after 6 h with this plasmid, applying the calcium chloride method [15]: a solution of 750 ng of plasmid diluted in a solution of 0.1 M calcium chloride in 2 × *N*-2-hydroxyethylpiperazine-*N'*-2-ethanesulfonic acid (HEPES) buffered saline were deposited in each well. To test anti-KIZ antibody, 48 h after transfection, COS-1 cells were fixed in 4% paraformaldehyde (PFA) for 5 min, permeabilized with 1× phosphate buffered saline (PBS) , 0.2% Triton X100 solution for 15 min, and then, intracellular staining was performed applying anti-KIZ rabbit (1:50) and anti-c-myc mouse (1:500) (11667149001, Roche, Basel, Switzerland) antibodies in 1 × PBS, 0.1% gelatine, 0.05% Tween 20, 0.1% bovine serum albumin (BSA). We pre-incubated the primary antibody solution with crude COS-1 cells (1:100) for 15 min at 37 °C and deposited this antibody solution on transfected cells for 1 h at room temperature (RT). Secondary antibody solution, donkey anti-rabbit conjugated with Alexa Fluor 488 and donkey anti-mouse with Cy3 (1:1000) in 1 × PBS, 0.1% gelatin, 0.05% Tween 20, 0.1% BSA, was subsequently incubated 30 min at RT. After fixation with 1% PFA for 5 min, nuclei were stained with DAPI (1:1000). Cells were then mounted with a mounting medium (Fluoromount-G, SouthernBiotech, Birmingham, AL, USA) using coverslips.

To test if the anti-KIZ antibody is indeed able to detect the respective protein by immunoblotting and to investigate if KIZ shows a different protein profile in fibroblasts of patients compared to control upon Western blot analysis, the following procedures were performed: confluent transfected and untransfected COS-1 cells and fibroblasts of the patient CIC01225 and control were washed in 1 × PBS and collected by scrapping. After a short centrifugation (800 rpm for 5 min), the pellet cells were resuspended in lysis buffer (50 mM Tris pH 7.5, 150 mM NaCl, 1% Triton-X100), incubated at 4 °C for 30 min with intermittent vortexing and sonication three times for ten-seconds bursts. After centrifugation at 14,000 × g for 10 min, 20 μg of the clarified lysates were loaded on an SDS-gel and visualized with the anti-c-myc-mouse antibody (1:500) (11667149001, Roche) or the anti-KIZ polyclonal rabbit antibody (1:1000) (SAB2700541, Sigma-Aldrich) and respective secondary anti- mouse and anti-rabbit HRP-antibodies (1:10,000) (Jackson Immuno Research) using standard Western blot protocols.

While the mouse anti-PLK1S1 antibody recognized a 76-kDa protein, the predicted size of the full-length protein [14], the human anti-KIZ antibody recognized a protein of approximately 100 KDa. It was argued that this discrepancy is probably due to the amino acid composition of KIZ rather than any posttranslational modification [16].

### 2.8. Human Fibroblasts Staining and Cilia Length Measurements

Fibroblasts of CIC01225, Family C (737) and a non-mutated control were derived from human skin biopsies and were cultured in a specific medium (DMEM, 10% FBS, 2% sodium pyruvate and 1% penicillin streptomycin; Life technologies, Carlsbad, CA, USA) or in the same medium without antibiotics, in a humidified 37 °C, 5% CO_2_ incubator. After two passages, we induced monocilia formation in these fibroblasts; cell cultures were deprived of fetal bovine serum for 24 h prior to fixation; and immunostaining experiments were carried out in triplicates and performed as described previously [9]. Briefly, anti-human KIZ (1:250) and anti-acetylated-α-tubulin (1:1000) antibodies were applied to human fibroblasts with subsequent standard secondary antibodies in addition to DAPI (1:1000) counterstaining. Cells were then mounted with a mounting medium (Mowiol preparation, Sigma) using coverslips.

Cilium length was measured using a software (ImageJ software, Bethesda, MD, USA) [17]. The mounted preparations were used to capture 10 fields of cells on the confocal microscope giving data for approximately 6 cilia per field of view.

### 2.9. Preparation of Mouse Retinas

Six-week-old C57BL/6JRj (Janvier, Genest Saint Isle, France) mice were euthanized by CO_2_ administration and cervical dislocation. Eyes were removed and prepared as follows: a hole just behind the ora serrata was made, and the eyeballs were placed in 4% (*w/v*) PFA in 0.12 M phosphate buffer, pH 7.2 for 1 h at RT. The lens was removed, and the eyecups were fixed again for 20 min in 4% PFA and cryoprotected with increasing concentrations of ice-cold sucrose in 0.12 M phosphate buffer, pH 7.2 (10% for 1 h and 30% overnight). Subsequently, the eyecups were embedded in 7.5% gelatin, 10% sucrose 1 × PBS and frozen in a dry ice-cooled isopentane bath. Subsequently, 10-μm retinal sections were made with a cryostat (model HM560; Thermo Fisher; Microm, Walldorf, Germany) [18], mounted with a mounting medium (Mowiol preparation, Sigma) on glass slides (Super-Frost, Thermo Fisher Scientific, Waltham, MA, USA) and kept at −80 °C.

### 2.10. PLKLS1 Localization in Mouse Retina

Mouse cryosections were post-fixed in methanol at −20 °C for 5 min and then blocked with 5% BSA, 0.1% Triton X-100, 0.1% heat-inactivated goat serum and 1 × PBS for 1 h at RT. An overnight incubation at 4 °C with anti-mouse PLK1S1 (1:500), anti-α-acetylated and anti-γ-tubulin (1:1000 was performed prior to washing 3 times for 30 min each in PBTrit (PBS and 0.1% Triton X-100). Donkey secondary antibodies anti-rabbit Alexa Fluor 488 (1:1000) and donkey anti-mouse Cy3 (1:1000) or donkey anti-mouse Alexa Fluor 647 (1:1000), DAPI (1:1000) and lectin PNA conjugate 594 (1:1000) were used during an overnight incubation at 4 °C [14]. Negative controls were performed with the use of secondary antibodies alone. Sections were washed 3 times for 30 min in PBTrit and then mounted with a mounting medium (Mowiol preparation, Sigma) using coverslips.

### 2.11. PLK1S1 Localization in Isolated Mouse Photoreceptors

Fresh mouse retinas were gently vortexed for 30 s in 400 μL of 1 × PBS with calcium; this step allows a separation of the photoreceptor sensory cilium (PSC) complexes from the remainder of the retina. The PSC complexes were collected from the upper fraction of the supernatant using a wide-open pipette transferred on a glass slide (Super-Frost, Thermo Fisher Scientific) and fixed with methanol at −20 °C for 5 min. After fixation, PSC were blocked in 0.2% gelatin, 0.1% Triton X-100 and in 1 × PBS for 1 h at RT. Staining was achieved following overnight incubation at 4 °C with anti-mouse PLK1S1 (1:250) and anti-rhodopsin (1:250), anti-mouse PLK1S1 (1:250) and anti-α-acetylated (1:500) or anti-γ-tubulin (1:1000). After incubation, PSC were washed twice in PBT (1 × PBS and 0.1% Tween 20) and stained with anti-rabbit Alexa Fluor 488 (1:1000) and anti-mouse Cy3 (1:1000) for 1 h at RT and then mounted with a mounting medium (Mowiol preparation, Sigma) on coverslips.

## 3. Results

### 3.1. Identification of Additional RCD Patients with Mutations in KIZ

**Figure 1:**
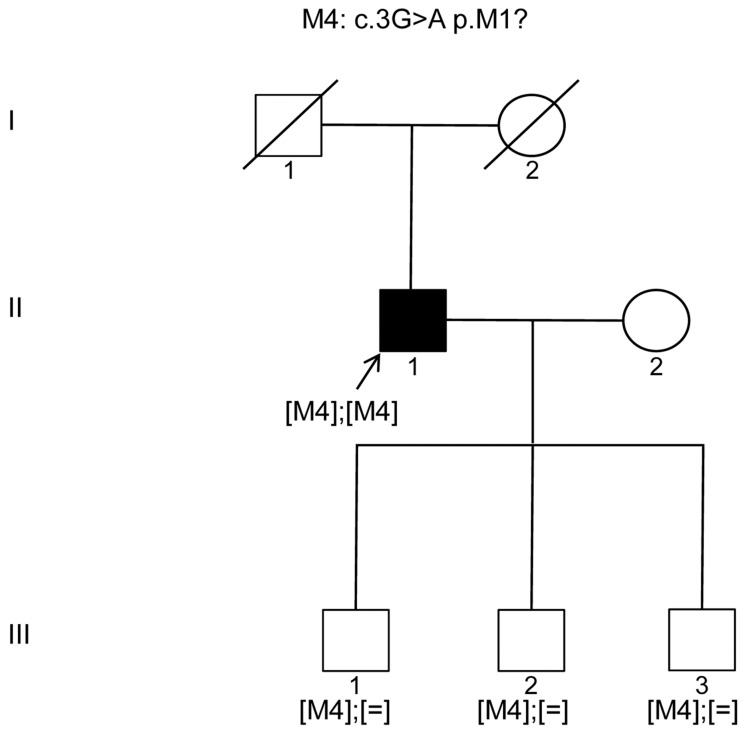
Pedigree of the Italian family with the novel *KIZ* mutation, c.3G>A p.M1?, underlying autosomal recessive rod-cone dystrophy. Shapes filled with black and white colors, respectively, represent affected and unaffected individuals. Squares and circles respectively indicate men and women. ↖ marks the index subject.

We report herein another patient (CIC07875 of family F4400) with RCD showing the known M1 *KIZ* mutation, c.226C>T (p.Arg76*), in a presumably homozygous state (Table 1) [9]. In absence of DNA of other family members, co-segregation studies could not be performed. The parents were reported to be cousins with Sephardic Jewish origin from Tunisia. Interestingly, one of the previously reported cases with the same mutation was also from North African Sephardic Jewish ancestry, and another one was of Spanish ancestry [9]. None of the three cases reported any awareness of a family connection. Therefore, we concluded this variant might be of Mediterranean origin. However, more recently, the same mutation was detected in two sisters with RCD from an Ashkenazi Jewish pedigree [19]. This population originates from Eastern and Central Europe, but also has Middle Eastern ancestry, which might reflect that this mutation cannot be assigned to a specific location, but represents a common cause. Indeed, this variant is now referenced showing a frequency in gnomAD (Table 1), but only at a heterozygous state in Ashkenazi Jews, Europeans and in Latinos with a global minor allele frequency <0.001 (109 from a total of 247,102 alleles) [13]. More interestingly, using targeted NGS, we detected a novel homozygous *KIZ* mutation, M4: c.3G>A (p.M1?), in an Italian index patient (Figure 1, Patient II.1). If the nucleotide exchange is translated, it would lead to an isoleucine instead of a methionine (p.M1I). This variant was shown to be rare, conserved in 27/30 species representing a coding sequence at this position, with three species showing another amino acid residue at this location, but never an isoleucine (Table 1). An isoleucine amino acid residue at this site was predicted to be disease causing (Polyphen and SIFT respectively), when we initially identified the mutation. However, due to mutation nomenclature recommendation, the mutation is better expressed as p.M1?. Since the starting methionine is affected, it is most likely that the protein will not be synthesized. Sanger sequencing confirmed the c.3G>A p.M1? variant, which co-segregated with the phenotype. While the index is most likely homozygous for the variant, the three unaffected siblings are carrying the heterozygous mutation (Figure 1, Subjects III.1, III.2 and III.3).

### 3.2. Expression of KIZ is Significantly Reduced in Whole Blood, but Not in Fibroblast Cells

**Figure 2:**
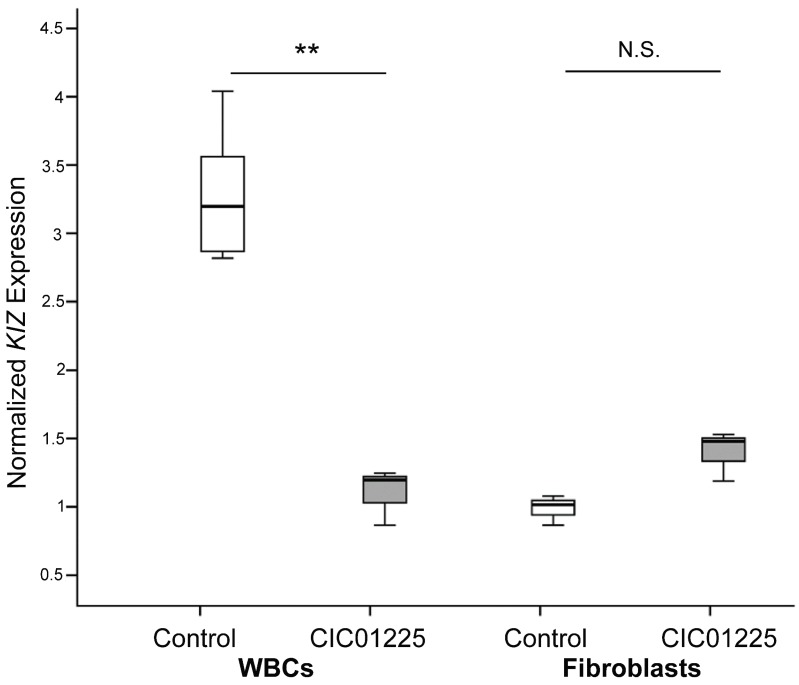
*KIZ* expression in whole blood cells (WBCs) and human fibroblasts of CIC01225 and in the non-mutated control. Quantitative real-time PCR, normalized to the expression of 18S, revealed higher expression in the control WBCs when compared to the ones of CIC01225 (** *p* ≤ 0.01). A 1.4-fold increase of *KIZ* expression has been observed in patient CIC01225 fibroblasts compared to control, which was however not significant. The data are presented in a box plot where in the median (bar), the maximum and minimum values and the quartiles are shown. N.S.: not significant. ** *p* < 0.01.

Real-time PCR analysis using primers in exons 6 and 7 of *KIZ* revealed mRNA levels in whole blood cells which were three-times lower in patient CIC01225 (compound heterozygous for the c.52G>T (p.Glu18*) and c.119_122del (p.Lys40Ilefs*14) mutations in *KIZ*) than in three controls (Figure 2, left). In contrast, a 1.4-fold increase of *KIZ* expression has been observed in patient CIC01225 fibroblasts compared to control (*p* = 0.051, Figure 2, right), which may reflect a trend, but which is statistically speaking only marginally significant. Especially, working with cells showing a certain variability of gene expression, we do not consider this slight change as of biological relevance. Subsequent Sanger sequencing of the PCR products confirmed the correct *KIZ* fragment amplification. We therefore predict that the previously reported compound heterozygous mutations consisting of a stop variant c.52G>T (p.Glu18*) and a frameshift deletion c.119_122del (p.Lys40Ilefs*14) may result in decreased *KIZ* gene expression in whole blood cells, but not in fibroblasts under our experimental conditions.

### 3.3. Transcript Analyses of KIZ in Whole Blood, Fibroblasts and Retina

All mutations identified so far are located in the first three exons of *KIZ* containing 13 exons (transcript 1, NM 018474.4). Patient CIC01225 from whom the expression of *KIZ* was investigated in blood and fibroblasts has a compound heterozygous mutation in exons 1 and 2. At least seven other isoforms of *KIZ* have been described (transcript 2, NM_001163022.2; transcript 3, NM_001163023.2; transcript 4, NM_001276389.2; transcript 5, NM_001352434.1; transcript 6, NM_001352435.1; and transcript 7, NM_001352436.1). Interestingly, all these other transcripts lack or reveal different exons (exon 3 is missing in transcript 2; exons 3 and 4 are missing in transcript 3; exons 2, 3 and 4 are missing in transcript 4; part of exon 7 is missing in transcript 5; exon 3 is missing in transcript 6; and a new exon between exons 3 and 4 is present in transcript 7).

**Figure 3:**
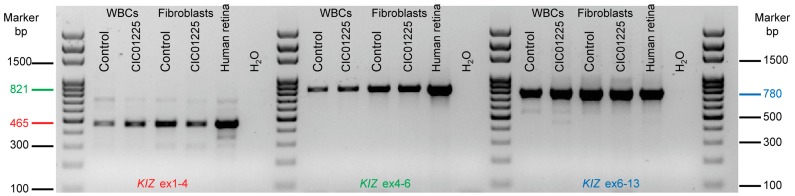
Presence of *KIZ* transcript 1 (NM 018474.4) in WBCs (whole blood cells), human fibroblasts of CIC01225 and in non-mutated control and human retina. PCR on cDNA revealed in all tested tissues exons 1–4 (red), exons 4–6 (green) and exons 6–13 (blue) transcripts with the expected size of 465, 821 and 780 bps, respectively. Sanger sequencing confirmed the correct sequences of the respective fragments and showed in addition the presence of the mutations in the cDNA made from WBSs and fibroblasts from the patient CIC01225.

In addition, in some isoforms, the predicted open reading frame is different and/or does not start in the first exon, which would affect the impact of *KIZ* variants described herein and previously reported [19]. To validate if transcript 1 (NM 018474.4) is present in blood, fibroblasts and human retina, reverse transcription-PCR (RT-PCR) experiments were performed. Indeed major bands could be detected in amplicons covering exons 1–4, exons 4–6 and exons 6–13 in all tissues tested, studies that were confirmed by Sanger sequencing in control and patient whole blood, fibroblast cells and control retina (Figure 3). Other transcripts may be also present, but due to the low abundance, we did not follow up on these amplicons (Figure 3).

### 3.4. An Anti-KIZ Antibody Detects the Human KIZ Protein in Mammalian Cells Overexpressing KIZ

**Figure 4:**
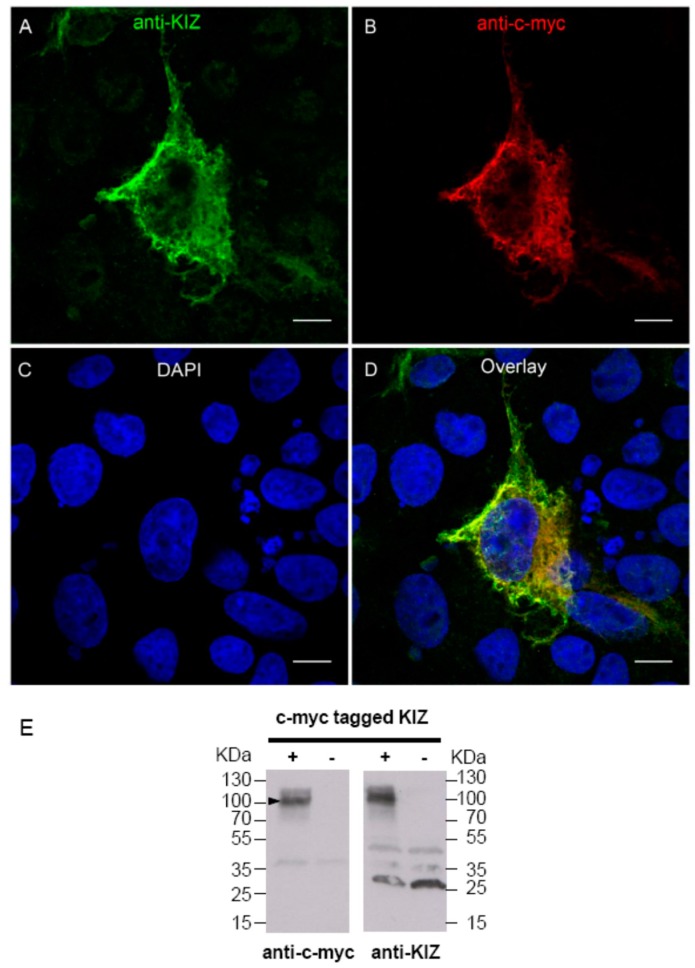
An anti-KIZ antibody is able to detect human KIZ in mammalian cells overexpressing c-myc-tagged KIZ. (**A**) The anti-KIZ antibody revealed intracellular staining (green) of c-myc-tagged human KIZ. (**B**) The anti-c-myc antibody revealed intracellular staining (red) of c-myc-tagged human KIZ. (**C**) Nuclei were stained with DAPI. (**D**) Merged staining reveals a costaining (yellow) indicating that both anti-KIZ and anti-c-myc antibodies detected the human KIZ in overexpressing COS-1 cells. Scale bar represents 10 µm. (**E**) The anti-c-myc and anti-KIZ antibodies detected a band at ~100 KDa (filled arrow head in protein extract of mammalian cells overexpressing c-myc-tagged KIZ (left and right blot). The band at ~100 KDa was absent in untransfected cells when stained with anti-c-myc (left blot) and anti-KIZ (right blot).

To perform functional analysis in human-derived fibroblast cells from patient and a control subject, a commercial anti-KIZ antibody was validated for its ability to detect the human KIZ protein in COS-1 cells overexpressing c-myc-tagged human KIZ. Indeed, intracellular staining revealed a co-localization of c-myc and KIZ, highlighting the ability of this antibody to detect the protein by immunolocalization studies (Figure 4A–D). To validate if the commercial anti-KIZ antibody is also able to detect the human KIZ protein in overexpressing cells by immunoblotting, Western blot analysis was performed. These studies showed a strong signal at ~100 KDa, which could be detected by the anti-c-myc (left panel), as well as by the anti-KIZ antibody (right panel) (independent Western blots), which was absent in untransfected cells (left and right panel), confirming that this antibody is able to detect the human KIZ protein (Figure 4E, filled arrow head depicted in the left panel). Although this antibody is able to detect human KIZ upon immunolocalization and Western blot analyses, other proteins not detected by the anti-myc antibody were also visible.

### 3.5. KIZ Localization, Monocilia Length and Protein Pattern Are Similar in Patient-Derived Fibroblasts When Tested with a Human KIZ Antibody

**Figure 5:**
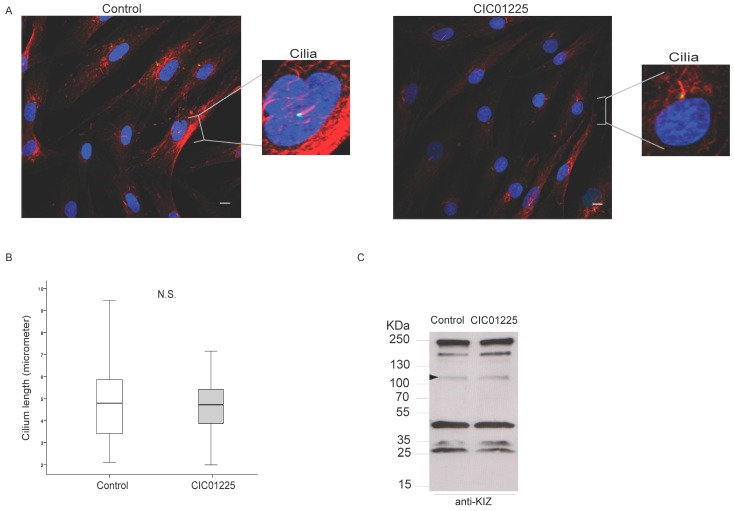
KIZ immunolocalization, cilia formation and immunoblot of human fibroblasts of non-mutated control and patient CIC01225 with the c.52G>T (p.Glu18*) and c.119_122del (p.Lys40Ilefs*14)) mutations. The data are presented in a box plot where in the median (bar) the maximum and minimum values and the quartiles are shown. (**A**) Fibroblasts were immunolabeled with anti-human KIZ antibody, anti-acetylated α-tubulin antibody and counterstained with DAPI. The overlay view shows co-localization of KIZ to the basal body of the cilium-associated centriole in the control and CIC01225. (**B**) Cilium length was measured in µm and then compared between the control (*N* = 76) and CIC01225 (*N* = 62), showing no significant difference. Scale bar represents 10 µm. (**C**) Immunoblot analyses of control and CIC01225 fibroblasts protein extracts stained with the anti-KIZ antibody showing the same profile with respect to band appearance and protein amount. Among others, a ~100-KDa band appeared (filled arrow head).

To determine whether the compound heterozygous *KIZ* mutations present in the patient CIC01225 (c.52G>T (p.Glu18*) and c.119_122del (p.Lys40Ilefs*14)) leading to an absence of protein and to test physical dysfunction of ciliogenesis in vivo, we performed immunolocalization studies in fibroblasts obtained from the patient CIC01225 and a control lacking any disease-causing mutation in *KIZ* (Figure 5A). Subsequently, the monocilia length of both subjects was measured (Figure 5B). KIZ was present at the base of the cilia in the control, but surprisingly, also in the fibroblasts from the patient (Figure 5A). In addition, the cilia length (Figure 5B) and the number of ciliated fibroblasts did not differ. Upon immunoblot analysis, the same profile with respect to the band appearance and protein amount was found in the protein extract from patient and a control (Figure 5C). Among others, a ~100-KDa band appeared (Figure 5C, filled arrow head).

### 3.6. PLK1S1 the Mouse Ortholog of KIZ Is Localized at the Basal Body of Mouse Photoreceptors

**Figure 6:**
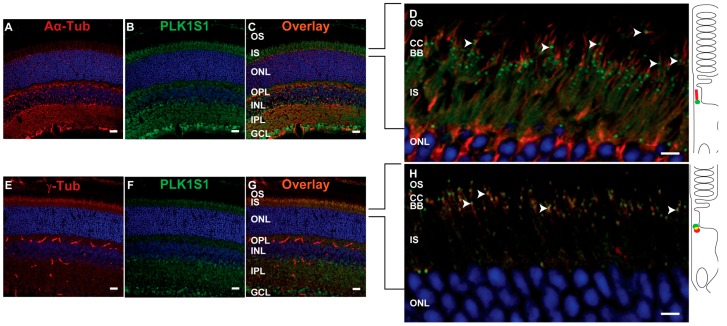
Immunolocalization of PLK1S1 in adult mouse retina. (**A**,**B**) Mouse retinal sections were stained with anti-acetylated α-tubulin (Aα-Tub) and anti-mouse PLK1S1 antibodies. (**C**) Merged images show that PLK1S1 is localized between the outer segments (OS) and inner segments (IS), in the outer plexiform layer (OPL), in the inner plexiform layer (IPL) and in the ganglion cell layer (GCL). (**D**) A close-up image of the OS and IS revealed PLK1S1 (arrow heads) staining in close vicinity to the base of acetylated α-tubulin (Aα-Tub) stripe, which resembles the connecting cilium. (**E**,**F**) Mouse retinal sections were stained with γ-tubulin (γ-Tub) and anti-mouse PLK1S1 antibodies. (**G**) Merged images show partial co-localization at the level of adjacent centriole of the basal body in photoreceptor cells. (**H**) A close-up image reveals a partial overlap (yellow) between PLK1S1 (arrow heads) and γ -tubulin. Nuclei are stained in DAPI (blue). Scale bar represents 20 µm (**A**–**C**,**E**–**G**) and 5 µm (**D**,**H**). α: alpha, CC: connecting cilium, γ: gamma, BB: basal body, ONL: outer nuclear layer. At the right of the close-up figures, a schematic drawing is depicted aiming to summarize the localization of the acetylated α-tubulin staining (red) and PLK1S1 (green) (upper drawing) and γ-tubulin (red) and PLK1S1 (green) (lower drawing).

Due to the limitations of fibroblasts to reflect immunolocalization of KIZ in the retina and further decipher pathophysiology, we studied the immunolocalization of the mouse ortholog of KIZ, PLK1S1 on fixed mouse retina cryosections using three different primary antibodies: anti-PLK1S1, anti-acetylated α-tubulin and anti-γ-tubulin (Figure 6). As mentioned in the Materials and Methods section, the ability of the anti-PLK1S1 antibody to detect the mouse protein had already previously been documented [14]. Whereas the anti-acetylated α-tubulin is a marker of stabilized microtubules in the ciliary axoneme [21], the anti-γ-tubulin antibody stains the adjacent centriole of the BB [21]. PLK1S1 immunostaining was found in different cell layers of the retina including between the outer and inner segments (OS and IS), in the outer plexiform layer (OPL), in the inner plexiform layer (IPL) and in the ganglion cell layer (GCL) (Figure 6B,C,F,G). Zooming into the OS and IS part of the retina revealed that PLK1S1 can be found at the base of acetylated α-tubulin (Figure 6D) and overlaps in its lower half with γ-tubulin (Figure 6H), indicating most likely its presence at the CC and BB.

**Figure 7:**
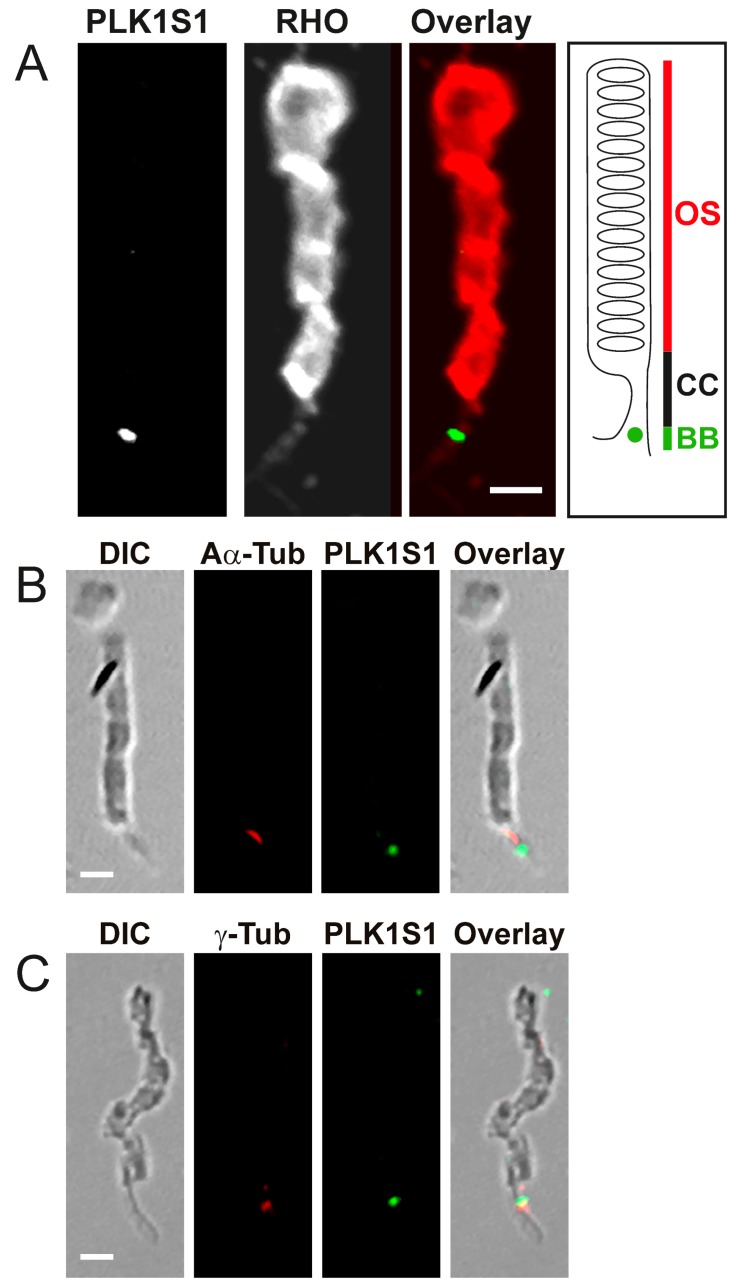
Immunolocalization of PLK1S1 in isolated mouse rod photoreceptors. (**A**) Dissociated photoreceptors were co-stained with antibodies against PLK1S1 (white or green in overlay) and against rhodopsin (white or red in overlay). PLK1S1 localizes at the base of OS most likely at the BB of the CC in dissociated mouse rod photoreceptors. Scale bar represents 2 µm. CC: connecting cilium. (**B**,**C**) Dissociated photoreceptors were visualized with differential interference contrast (DIC) and co-stained with (**B**) anti-acetylated α-tubulin (Aα-Tub) (red) or (**C**) γ-tubulin (γ-Tub) (red) and PLK1S1 (green) confirming the BB localization of PLK1S1 in rod photoreceptors. Scale bar represents 2 µm.

Subsequently, we performed a mechanical dissociation of mouse retinas, allowing rod photoreceptor isolation with preservation of OS, CCs and sometimes a small part of the IS. Double staining of RHO and PLK1S1 revealed a specific localization of PLK1S1 at the basal body complex of the connecting cilium of rod photoreceptors, a region located between the IS and the OS (Figure 7A). Co-immunolocalization studies on isolated photoreceptors with anti-acetylated α-tubulin and anti-γ-tubulin antibody revealed PLK1S1 staining close to the anti-acetylated α-tubulin staining (Figure 7B) partly overlapping with anti-γ-tubulin (Figure 7C), indicating that PLK1S1 is located at the BB in the CC (Figure 7).

**Figure 8:**
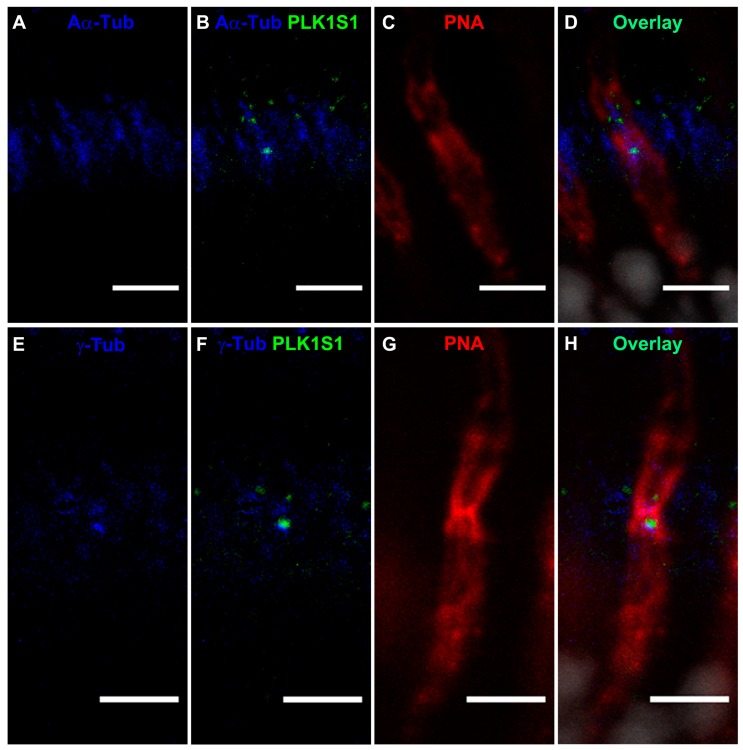
Immunolocalization of PLK1S1 in adult mouse retina revealed PLK1S1 localization in the BB of cone photoreceptors. (**A**–**H**) Close-up of cilia (acetylated α-tubulin (Aα-Tub) or γ-tubulin (γ-Tub), blue) of the cone photoreceptor (PNA, red). Merged images show that PLK1S1 is localized in close vicinity of acetylated α-tubulin and γ-tubulin most likely in the basal body of cone photoreceptors. Nuclei are stained in DAPI (gray). Scale bar represents 5 µm.

Interestingly, co-staining images of PLK1S1 (Figure 8B,D,F,H), cone photoreceptors stained with PNA (Figure 8C,G) and cilia proteins stained with anti-acetylated α-tubulin (Aα-Tub) (Figure 8A,B,D) and γ-tubulin (γ-Tub) antibodies (Figure 8E,F,H) in adult mouse retina revealed that PLK1S1 is most likely also localized in cone photoreceptors, presumably at the BB of the CC (Figure 8).

## 4. Discussion

We recently identified three different mutations in *KIZ*; c.226C>T (p.Arg76*), c.119_122del (p.Lys40Ilefs*14) and c.52G>T (p.Glu18*) in three patients with arRCD [9]. *KIZ* codes for a 673-amino acid protein representing a centrosomal substrate of Polo-like kinase-1 that mediates mitotic chromosomal stabilization [16]. In 2008, Tang and co-workers [14] reported that the mouse ortholog PLK1S1 (called Gm114 at this time) is expressed in male germ cells at early stages of embryogenesis, in spermatocytes and in spermatids of adult mouse testis, suggesting an important role in mammalian germ line stem cell self-renewal and differentiation [14]. The role of KIZ or its mouse ortholog PLK1S1 in retina function was less well understood. Quantitative real-time PCR in human tissues showed that the *KIZ* transcript was most abundant in the retina, followed by the retinal pigment epithelium, whole blood cells and fibroblasts. Immunofluorescent staining of serum-starved human fibroblasts showed KIZ immunolocalization to the basal body of monocilia [9]. Additional in silico transcriptomic analyses in mice revealed that the mouse ortholog *Plk1s1* is expressed in rod photoreceptors and ex in vivo studies showed its expression in the outer nuclear layer of the mouse retina [9]. Herein, we show that PLK1S1 protein is also localized in the mouse retina and more precisely at the BB of the CC in rod photoreceptors, but most likely also in cone photoreceptors. Due to their presence on a wide variety of mammalian cells (including human), defects in cilia metabolism are associated with an increased number of Mendelian and complex diseases [22]. These comprise a heterogeneous group of pathologies including inherited retinal diseases [4], cystic kidney disease [23], infertility [24], chronic respiratory problems, hypertension [25] and obesity [25]. It is remarkable that half of the implicated genes encoding ciliary proteins are localized in the BB of photoreceptors. This observation underlies the necessity of maintaining an adequate bridge-like structure required for a correct trafficking process and serving as a gate for proteins destined for the ciliary transport [26]. Since the CC, of which the core of the cilium extends from the BB, is the only intracellular passage between the IS and OS of the photoreceptors [26], all molecules have to traffic from the synthesis location within the cell body and the IS through the CC to reach the OS. This trafficking (import/export) is thought to be regulated by proteins arranged in networks at the BB [2]. The localization of PLK1S1 at the BB suggests its putative involvement in the regulation of this complex transport network.

Our studies herein revealed two additional patients with most likely homozygous mutations in *KIZ*; one patient harbored the previously identified mutation c.226C>T (p.Arg76*) [9] and the other one the c.3G>A (p.Met1?) affecting presumably the start codon of *KIZ*. Recently, the same c.226C>T (p.Arg76*) mutation was detected in two sisters with RCD from an Ashkenazi Jewish pedigree [19]. To our knowledge, our work on *KIZ* mutations is only the third report documenting mutations in arRCD patients. This might be due to the fact that the prevalence of this gene defect seems to be rare and perhaps only a few research groups have added this gene on their targeted NGS panel. However, our study highlights the importance of adding this gene to such high throughput screening panels especially when screening Ashkenazi Jewish and Latino populations.

Furthermore, our studies showed that human fibroblasts derived from patients carrying truncating mutations in *KIZ* are most likely not a good model to study the pathogenic mechanism of this gene defect. This holds true for expression, protein localization and functional analyses. In most cases, mutations leading to a premature termination codon tend to trigger nonsense-mediated mRNA decay (NMD); however, in other cases, NMD remains inactive, resulting in a truncated protein with a premature termination codon. Albeit we showed that *KIZ* expression in whole blood cells was three-times lower in a patient with truncating mutations compared to controls, no significant difference of *KIZ* expression was detected in mutant *KIZ* patient-derived fibroblasts compared to controls. Since *KIZ* mRNA levels in fibroblasts did not significantly differ between the affected individual and the control, we speculate that the phenomenon of NMD is not active in this tissue under the in vitro condition we used. Interestingly, our previous expression studies revealed already that *KIZ* expression is quite low in fibroblasts < blood < RPE < retina with the highest expression in retina [9], further confirming the possibility that fibroblasts are most likely not the best in vitro system to study the pathogenic mechanism of mutations in *KIZ*. This might be due to tissue-specific transcripts. For *KIZ*, seven different transcripts have been described of which only transcript 1 (NM 018474.4) and transcript 5 (NM_001352434), of which part of exon 7 is missing, are predicted to be affected by the mutations found in exons 1 and 2 in the studied patient. To investigate, if tissue specific transcripts may account for the unaltered *KIZ* expression observed in patient fibroblasts compared to control, the presence of transcript 1 was analyzed in blood, fibroblasts and retina. All tissues revealed indeed transcript 1 at high expression levels as shown by RT-PCR experiments, arguing against the hypothesis that in fibroblasts transcript 1 is not present and, thus, the mutations do not affect the expression. However, we cannot exclude that other transcripts are also present albeit at lower expression levels, rescuing the phenotype in fibroblasts. At the same time, in order to investigate the function and pathogenic mechanism on protein level in human fibroblasts, immunolocalization studies, cilia length measurements and immunoblot analyses were performed at the same time with a commercially available antibody. This antibody directed against the human KIZ protein revealed immunolabeling and a band at ~100 KDa upon Western blot analyses in cells overexpressing a tagged protein, which was absent in untransfected cells documenting that this antibody can indeed detect KIZ in this system. Immunolabeling and a band at ~100 KDa upon Western blot in HeLa cells, which could be blocked by small interfering RNA-treated cells, had also previously been reported for a different KIZ antibody [16]. However, when we applied the antibody that we checked in overexpressing cells, we could detect KIZ not only in control’s, but also in patient's fibroblasts, localizing it at the basal body of monocilia. In addition, cilia length did not differ between case and control samples (*p* > 0.05). Similarly, the same protein profile in patient- and control-derived fibroblast cells upon immunoblotting was detected showing among others a band at ~100 KDa. Mass spectrometry investigation of the band needs to be done to confirm if this band represents indeed human KIZ.

Therefore, the question remains, why KIZ would be still present in patient fibroblasts albeit the truncating mutations. NMD was first described in 1979 in human cells and in yeast as implicated in eliminating truncated proteins [27]. In mammalian cells, its main function is to reduce errors in gene expression by eliminating mRNA transcripts that contain premature termination codons [28] at more than 50–55 bp upstream of an exon-exon junction. Mechanistic studies reported that NMD efficiency can vary between cell types [29], tissue types [30] and individuals [31]. Furthermore, it was also shown that nonsense mutations in the first exon of certain genes (such as *Beta Globin*) do not activate NMD [32,33] since essential NMD components cannot be recruited. According to the 50–55-bp rule, both c.52G>T, p.Glu18* and c.119_122del, p.Lys40Ilefs*14 in KIZ may escape the NMD process as they are less than 50 bp from the exon-exon junction. Interestingly, c.52G>T, p.Glu18* mutation is located in the first exon of KIZ. However, if these mutants escape NMD, this would result in very short proteins, most likely not detectable with the anti-KIZ antibody, directed against the C-terminal region of the protein.

On the other hand, a premature termination codon read-through could occur in fibroblasts from patient and control, subsequently restoring a full-length protein in patient’s cells, especially since our fibroblast cells were initially cultured in a media containing 1% of aminoglycosides antibiotics (see Materials and Methods, e.g., streptomycin). This hypothesis is supported by the work of Schwarz and co-workers [34] on induced pluripotent stem cells (iPSC)-derived retinal pigment epithelium cells from an Xl RCD patient, which showed that aminoglycosides (G418) and other molecules (PTC124) can promote read-through of the RP2 p.Arg120* mutation and thus restore *RP2* function [34]. However, when we performed these studies using aminoglycoside-free media, we could not see any difference with respect to protein localization, cilia length and protein expression between mutant and control cells.

An alternative explanation would be that other isoforms, not affected by the two mutations found in the studied patient, but also detected by the antibody are still formed in fibroblasts, and thus, the phenotype is not observed in this system. Indeed the anti-KIZ antibody is a rabbit polyclonal antibody produced with a recombinant human KIZ fragment corresponding to the region between amino acids 187 and 438 of the human protein. These amino acids are present in all seven isoforms. Again, if indeed other transcripts are present in addition to transcript 1, albeit at lower levels and the respective mRNA translated, the respective proteins could be detected by this antibody. This finding urges for a specific antibody directed against isoform 1.

Although, we did not detect any physical effect on ciliogenesis in fibroblasts, we cannot exclude that mutations in *KIZ* affect the cilia structure or function in the human retina itself. Despite the presence of cilia in a variety of tissues [35], mutant ciliary proteins may have various impacts on their stability or their consequences may be variable in maintaining cellular homeostasis among these different tissues [36,37]. This variability may explain the restricted retinal phenotype found in our RCD patients carrying *KIZ* mutations [9] and other cases of isolated retinopathies linked to mutations on other ciliary proteins [38].

Future directions would be to generate human pluripotent stem cells from fibroblasts with subsequent differentiation in retina-like structure in vitro [39]. Such a step may provide a more relevant in vitro model of the disease and unravel the molecular and cellular mechanisms associated with mutations in *KIZ* and other ciliary proteins underlying RCD.

While the present study provides new insights into the molecular properties of KIZ and its mouse ortholog, future in vivo (animal models) and in vitro (human pluripotent stem cells) studies will help to understand its precise role in the retina and the associated pathogenic mechanism(s) leading to this form of RCD.

## Figures and Tables

**Table 1:** Novel patients with rod-cone dystrophy and *KIZ* mutations.

Patient and Family	Exon	Nucleotide Exchange	Protein Exchange	Mutation	dbSNP	gnomAD	ESP	Reference
				Name				
CIC07875, F4400	3	c.226C>T	p.R76*	M1	rs202210819	ALL: T = 0.041%; Ashkenazi Jews = 0.7%; Latino = 0.1%; African: 0.0045%- European = 0.006%; East Asians = 0%; Finnish = 0%; Other: 0.05%	EA: T = 0.06%; AA: T = 0.00%	[9]
Italian patient	1	c.3G>A	p.M1?	M4	-	ALL: A = 0.00091%; Ashkenazi Jews = 0%; Latino = 0.005%; African: 0%; European = 0.%; East Asians = 0%; Finnish = 0%; Other: 0.05%	-	novel

Mutation annotation and prevalence data was taken from a software (Alamut, v2.7-1, Interactive Biosoftware, Rouen, France) using *KIZ* (NM_01874.4) and human mutation recommendation [20]. gnomAD = Genome Aggregation Database, ESP = Exome Sequencing Project.
